# *In vitro* study on α-amylase inhibitory activity of an Indian medicinal plant, *Phyllanthus amarus*

**DOI:** 10.4103/0253-7613.70107

**Published:** 2010-10

**Authors:** Iniyan G. Tamil, B. Dineshkumar, M. Nandhakumar, M. Senthilkumar, A. Mitra

**Affiliations:** Department of Bio-technology, Prathyusha Institute of Technology and Management, Chennai - 602 025, India; 1 Department of Bio-technology, School of Medical Science and Technology, IIT Kharagpur, Kharagpur - 721 302, India

**Keywords:** *Phyllanthus amarus*, extracts, pancreatic amylase inhibitory effects

## Abstract

**Objective:**

The objective of this study was to evaluate the α-amylase inhibitory activity of different extracts of *Phyllanthus amarus* against porcine pancreatic amylase *in vitro*.

**Materials and Methods:**

The plant extracts were prepared sequentially with ethanol, chloroform, and hexane. Each extract was evaporated using rotary evaporator, under reduced pressure. Different concentrations (10, 20, 40, 60, 80, and 100 μg/mL) of each extract were made by using dimethyl sulfoxide (DMSO) and subjected to α-amylase inhibitory assay using starch azure as a substrate. The absorbance was read at 595 nm using spectrophotometer. Using this method, the percentage of α-amylase inhibitory activity and IC_50_values of each extract was calculated.

**Results:**

The chloroform extract failed to inhibit α-amylase activity. However, the ethanol and hexane extracts of *P. amarus* exhibited appreciable α-amylase inhibitory activity with an IC50 values 36.05 ± 4.01 μg/mL and 48.92 ± 3.43 μg/mL, respectively, when compared with acarbose (IC_50_value 83.33 ± 0.34 μg/mL).

**Conclusion:**

This study supports the ayurvedic concept that ethanol and hexane extracts of *P. amarus* exhibit considerable α-amylase inhibitory activities. Further, this study supports its usage in ethnomedicines for management of diabetes.

## Introduction

Diabetes is a group of metabolic diseases characterized by hyperglycemia resulting from defects in insulin secretion. Food habits and genetic factors are responsible for diabetes. A study revealed that urbanization of rural India has doubled the rate of diabetes.[1] In India, between 1995 and 2025, the number of people with diabetes is projected to rise from 19 to 57 million. As per the National Urban Diabetic Survey, the incidence of diabetes was found to be high (Hyderabad 16.6%, Chennai 13.5%, Bangalore 12.4%, Kolkata 11.7%, Delhi 11.6%, and Mumbai 9.3%). Of all these diabetic populations, 80% account for Type 2 diabetes.[2] Clinical studies on different species of animals have shown that consuming less food (caloric restrictions) reduces the risk of diabetes and heart disease. Current treatment for Type 2 diabetes remains inadequate, prevention is preferable.[3] One therapeutic approach for treating Type 2 diabetes is to decrease postprandial hyperglycemia. Modern medicines such as biguanides, sulfonylureas, and thiozolidinediones are available for the treatment of diabetes. However, they also have undesired effects associated with their uses.[4] Alternative medicines, predominantly herbal drugs are available for the treatment of diabetes. Common advantages of herbal drugs are effectiveness, safety, and acceptability.[5] The medicinal plants or natural products involve retarding the absorption of glucose by inhibiting the carbohydrate hydrolyzing enzymes, such as pancreatic amylase. The inhibition of this enzyme delay carbohydrate digestion and protract overall carbohydrate digestion time, resulting in the reduction in glucose absorption rate and consequently dulling the postprandial plasma glucose rise. Several indigenous medicinal plants have a high potential in inhibiting α-amylase enzyme activity.[6] *Phyllanthus amarus* plant have been used predominantly in folk medicine worldwide for the treatment of various diseases such as jaundice, constipation, diarrhea, kidney ailments, ringworm, ulcers, malaria, genitourinary infections, hemorrhoids, and gonorrhea.[7,8] Studies showed that this plant has potential antiviral property against hepatitis B virus.[9] This study was carried out to evaluate *in vitro* inhibitory effect of various extracts (ethanol, chloroform, and hexane) of *P. amarus* on porcine pancreatic amylase activity.

## Materials and Methods

### Materials

Starch azure, porcine pancreatic amylase, and Tris–HCl were purchased from Sigma Aldrich, India. Hexane, chloroform, ethanol, dimethyl sulfoxide (DMSO), and acetic acid were purchased from Merck, India.

### Plant Materials

*Phyllanthus amarus* young whole plants were collected from the Villupuram district of Tamilnadu, India in the months of July–September 2007. The plant was botanically identified and authenticated by Dr. M. Senthilkumar, Plant Biotechnologist, University of Madras, Chennai, India. The herbarium of the plant was deposited in the PITAM against voucher no PITAM/CH/00012/2007. The plants were dried at ambient temperature (30–40°c) for 25–30 days. The dried plants were ground into fine powder using a domestic electric grinder [Product: (GX 21), Bajaj Appliances, Mumbai, India].

### Preparation of Plant Extracts

The dried powdered of whole plant of *P. amarus* was extracted using a soxhlet apparatus sequentially with ethanol, chloroform, and hexane. Each extracts were evaporated using rotary evaporator (Buchi R-210), under reduced pressure. The percentage yield of ethanol, chloroform, and hexane were 3.56%, 3.16%, and 1.48%, respectively. The dried extracts were dissolved in DMSO to make different concentrations, and subjected to α-amylase inhibitory assay.

### In vitro α-Amylase Inhibitory Assay

The assay was carried out following the standard protocol with slight modifications.[10] Starch azure (2 mg) was suspended in 0.2 mL of 0.5M Tris–HCl buffer (pH 6.9) containing 0.01 M CaCl_2_ (substrate solution). The tubes containing substrate solution were boiled for 5 min and then preincubated at 37°C for 5 min. Ethanol extract of *P. amarus* was dissolved in DMSO in order to obtain concentrations of 10, 20, 40, 60, 80, and 100 μg/mL. Then, 0.2 mL of plant extract of particular concentration was added to the tube containing the substrate solution. In addition, 0.1 mL of porcine pancreatic amylase in Tris–HCl buffer (2 units/mL) was added to the tube containing the plant extract and substrate solution. The reaction was carried out at 37°C for 10 min. The reaction was stopped by adding 0.5 mL of 50% acetic acid in each tube. The reaction mixture was centrifuged at 3000 rpm for 5 min at 4°C. The absorbance of resulting supernatant was measured at 595 nm using spectrophotometer (Perkin Elmer Lambda 25 UV–VIS spectrophotometer). Same procedure was followed for other plants extracts (chloroform and hexane) to test their α-amylase inhibitory effects. Acarbose, a known α-amylase inhibitor was used as a standard drug. The experiments were repeated thrice. The α-amylase inhibitory activity was calculated by using following formula:

$$\;\mathrm{The}\;\mathrm{\alpha -amylase}\;\mathrm{inhibitory}\;\mathrm{activity}\;=\;\left(\mathrm{Ac}+\right)\;-\;\left(\mathrm{Ac}-\right)\;-\;\left(\mathrm{As}\;-\;\mathrm{Ab}\right)/\left(\mathrm{Ac}+\right)\;-\;\left(\mathrm{Ac}-\right)\;\times \;100,$$

where Ac+, Ac−, As, and Ab are defined as the absorbance of 100% enzyme activity (only solvent with enzyme), 0% enzyme activity (only solvent without enzyme), a test sample (with enzyme), and a blank (a test sample without enzyme), respectively. The concentration of acarbose and plant extracts required to inhibit 50% of α-amylase activity under the conditions was defined as the IC_50_ value. The α-amylase inhibitory activities of plant extracts and acarbose were calculated, and its IC_50_ values were determined.

### Statistical Analysis

All values were expressed mean ± SD. Statistical difference and linear regression analysis were performed using Graphpad prism 5 statistical software.

## Results

**Figure 1:**
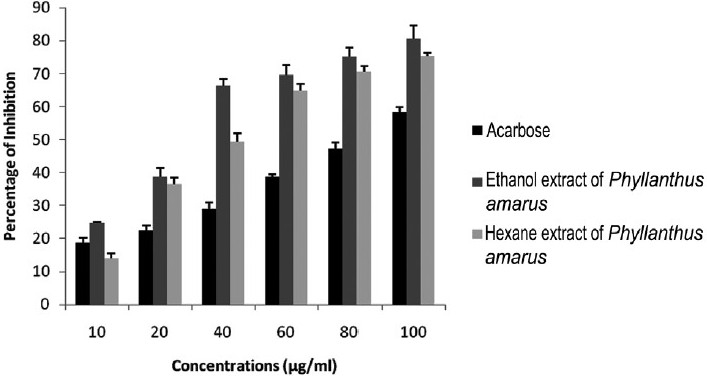
Percentage of α-amylase inhibitory effects of acarbose (standard drug), ethanol, and hexane extracts of *P. amarus*.

Acarbose (at a concentrations 100 μg/mL) showed 58.45% inhibitory effects on the α-amylase activity with an IC_50_ value 83.33 ± 0.34 μg/mL [Table 1]. The ethanol extracts of *P. amarus* (at a concentration 100 μg/mL) exhibited 80.48% of α-amylase inhibitory activity with an IC_50_ values 36.05 ± 4.01 μg/mL. The hexane extracts of *P. amarus* (at a concentration 100 μg/mL) exhibited 75.32% of α-amylase inhibitory activity with an IC_50_ values 48.92 ± 3.43 μg/mL [Table 2]. However, the chloroform extract did not show α-amylase inhibitory activity. Both ethanol and hexane extracts of *P. amarus* showed appreciable α-amylase inhibitory effects when compared with acarbose [Figure 1].

**Table 1:** Alpha-amylase inhibitory effects of acarbose (standard α-amylase inhibitor)

*Drug*	*Concentration (μg/mL)*	*% of Inhibition*	*IC_50_ value (μg/mL)*
Acarbose	10	18.75	83.33 ± 0.34
	20	22.41	
	40	29.06	
	60	38.74	
	80	47.27	
	100	58.45	

**Table 2:** Alpha-amylase inhibitory effects of ethanol and hexane extracts of *P. amarus*

*Plant extracts*	*Concentration (μg/mL)*	*% of Inhibition*	*IC_50_ value (μg/mL)*
Ethanol	10	24.76	36.05 ± 4.01
	20	38.61	
	40	66.46	
	60	69.51	
	80	74.99	
	100	80.48	
Hexane	10	13.09	48.92 ± 3.43
	20	36.48	
	40	49.33	
	60	64.82	
	80	70.60	
	100	75.32	

## Discussion

Many herbal extracts have been reported to have antidiabetic activities and are used in Ayurveda for the treatment of diabetes. Herbal extracts have been used directly or indirectly for the preparation of many modern medicines. In this study, an *in vitro* inhibitory effect of different extracts of *P. amarus* on porcine pancreatic amylase activities was evaluated. Antidiabetic activities of *P. amarus* have been reported with its leaves and seeds. The aqueous extracts of the leaves and seeds of *P. amarus* were successively tested in Type 1 diabetes *in vivo* in doses ranging from 150 to 600 mg/kg/day for 21 days. These had lowered the blood glucose level successively, which may be due to the increased level of insulin in the blood.[11] Another previous study had reported the antidiabetic property with the whole plant in Type 2 diabetes.

We compared IC_50_ values of α-amylase inhibitory effects of ethanol and hexane extracts of *P. amarus* with results obtained in previous studies. Hexane and dichloromethane extracts of *P. amarus* were successively studied using non-preincubation method (time-dependent ranging from 0 to 3 min) at 20 μg/mL concentration. However, the hexane extract had only showed significant activity with 24.3% α-amylase inhibition at 3 min.[12] In our study, the hexane extract (at a concentration 100 μg/mL) showed 75.32% of α-amylase inhibitory activity with IC_50_ value 48.92 μg/mL. At the same time, both ethanol and hexane extracts showed appreciable α-amylase inhibitory effects when compared with acarbose. It may be due to the presence of more chemical constituents such as lignans (phyllanthin and hypophyllanthin), terpenes, tripenes, flavonoids (quercetin, quercetrin, rutin), and alkaloids in the ethanol and hexane extracts. The plant-based α-amylase inhibitor offers a prospective therapeutic approach for the management of diabetes.[13] In this study, whole plants of *P. amarus* showed considerable α-amylase inhibitory effects when compared with acarbose.

## Conclusion

The results of study indicate that ethanol and hexane extracts of *P. amarus* plant showed appreciable α-amylase inhibitory effects. This study supports the ayurvedic concept that *P. amarus* could be useful in management of diabetes.
